# Temporal Patterns of Adverse Events Associated With Selective Serotonin Reuptake Inhibitors: A Global Pharmacovigilance Analysis of Early-Onset Versus Late-Onset Toxicity

**DOI:** 10.7759/cureus.100644

**Published:** 2026-01-02

**Authors:** Adrian Chin Yan Chan

**Affiliations:** 1 Pharmacology, Bayer Pharmaceuticals, Beijing, CHN

**Keywords:** adverse events, pharmacovigilance, selective serotonin reuptake inhibitors, time-to-onset, who vigibase

## Abstract

Background

Selective serotonin reuptake inhibitors (SSRIs) are commonly used as first-line antidepressant medications, but adverse events (AEs) remain a common reason for treatment discontinuation. Understanding when these AEs occur can help improve patient care and treatment adherence.

Objective

This study aims to explore the timing of AEs associated with six commonly used SSRIs: sertraline, fluoxetine, fluvoxamine, paroxetine, citalopram, and escitalopram using global pharmacovigilance data, with a focus on early- versus late-onset profiles.

Methods

This study analyzed Individual Case Safety Reports (ICSRs) from VigiBase, the World Health Organization's global safety database. Reports were included if an SSRI was the suspected drug and time-to-onset (TTO) data were available. AEs with at least 10 reported TTOs were grouped as early-onset (TTO ≤28 days) or late-onset (TTO >28 days).

Results

A total of 1,428 AEs met the inclusion criteria. Of these, 914 (64%) were early onset, including nausea (median TTO: 1 day), insomnia and dizziness (2 days), and sexual dysfunction (16.5 days). Late-onset AEs, 514 (36%), included weight gain (31 days), hyperhidrosis (76.5 days), diabetes mellitus (151 days), and osteoporosis (959.5 days). Early AEs were mostly gastrointestinal, neurological, or activation-related; late AEs were largely metabolic or endocrine.

Conclusions

SSRIs show distinct temporal AE patterns. Early-onset symptoms require timely management to improve tolerability, while late-onset effects highlight the need for ongoing monitoring. These findings can inform personalized monitoring strategies and guide patient counseling to support safer long-term SSRI use.

## Introduction

Selective serotonin reuptake inhibitors (SSRIs) are commonly used as first-line antidepressant medications worldwide and are widely utilized to treat depressive and anxiety disorders. While their efficacy is well established, adverse events (AEs) are frequently reported, contributing to treatment discontinuation in both randomized trials and real-world practice [1]. These AEs often vary in their temporal presentation, with some emerging early in treatment (e.g., gastrointestinal symptoms, activation, insomnia) and others manifesting later (e.g., weight gain, sexual dysfunction) [2,3]. Understanding the timing and characteristics of these AEs is therefore critical for optimizing clinical management and improving patient outcomes.

Existing AE data from patients receiving SSRIs are obtained primarily from controlled settings of randomized trials, where sample sizes and follow-up durations are limited, and patients are identified based on stringent inclusion and exclusion criteria that are not representative of real-world clinical populations. Additionally, although some studies and/or surveys have been conducted in a real-world setting that attempt to further understand the nature and temporal characteristics of AEs associated with SSRI therapy [3,4], these studies are also often limited by their small sample sizes and limited follow-up duration. Hence, there exist substantial gaps in our knowledge regarding the temporal characteristics and real-world safety profile of SSRI agents.

To address this gap, this study leverages cumulative AE data from VigiBase for six commonly prescribed SSRIs (sertraline, fluoxetine, fluvoxamine, paroxetine, citalopram, and escitalopram) to systematically examine the time-to-onset (TTO) of these AEs. VigiBase is the World Health Organization (WHO) global database of individual case safety reports (ICSRs), which is based on a large cohort of patients globally and provides a comprehensive and globally representative perspective on the real-world safety profiles of SSRIs.

This study aims to characterize the median TTO and clinical profile of SSRI-associated AEs, identify temporal patterns in AE emergence, and provide evidence-based guidance for clinicians to personalize monitoring and improve patient counseling. These findings may support more informed decision-making and enhance adherence by anticipating and managing adverse effects throughout the course of treatment.

## Materials and methods

Data source

This observational study utilized ICSRs from VigiBase, the WHO's global pharmacovigilance database maintained by the Uppsala Monitoring Centre (UMC). VigiBase is the largest international repository of spontaneous adverse drug reaction (ADR) reports, aggregating submissions from healthcare professionals, patients, and regulatory authorities across more than 130 countries [5]. Its extensive geographic coverage and long-standing operation provide a unique opportunity to examine real-world safety profiles of widely used pharmacological agents, including SSRIs.

SSRIs represent the most commonly prescribed class of antidepressants globally and are extensively used across diverse age groups and clinical settings in both high- and low-income countries [6,7]. However, because VigiBase relies on spontaneous reporting, the frequency of reported AEs does not reflect true incidence. Reporting is influenced by multiple factors, including prescribing volume, reporter awareness, perceived seriousness of events, regulatory activity, and media or scientific attention. It is well established that spontaneous reporting systems capture only a small fraction of actual AEs, with under-reporting estimates commonly ranging from approximately 1% to 10% of true events across pharmacovigilance systems worldwide [6,7]. Consequently, AE data in VigiBase should be interpreted as indicators of reporting patterns and temporal associations rather than absolute risk estimates. All AEs were coded using the Medical Dictionary for Regulatory Activities (MedDRA) Preferred Terms (PTs), ensuring standardized terminology and consistency across reports.

We focused on ICSRs involving six SSRIs: sertraline, fluoxetine, fluvoxamine, paroxetine, citalopram, and escitalopram, which are among the most frequently prescribed antidepressants in clinical practice. SSRIs account for the majority of antidepressant prescriptions worldwide across diverse populations, and recent real-world prescribing data indicate high utilization of this class in both primary and specialist care settings [6,7].

Data preparation

A cumulative retrieval from Vigibase of all ICSRs reporting any of the six SSRIs listed above as suspect medications was implemented, with a data lock point of 08 June 2024. The ICSRs were then aggregated across six SSRIs into a unified dataset. Individual drug names were removed, considering all SSRIs as a single class. Reports were included irrespective of the documented causality assessment in VigiBase (i.e., reports were not limited to "certain," "probable," or "possible" designations according to WHO-UMC or Naranjo criteria). This approach reflects typical pharmacovigilance practices in large spontaneous reporting analyses, where individual causality assessments are not uniformly available and are often incomplete, and where inclusion of all suspect reports enables comprehensive characterization of AE patterns. However, reports lacking essential fields (e.g., drug start date or event onset date) were excluded during TTO calculation because accurate temporal information is necessary for time-to-onset analyses. AEs with negative TTO values or missing TTO information were excluded. If an ICSR reports more than one valid TTO value for a particular AE term (i.e., the same AE was reported with valid TTO values more than once in a single patient), then only the smallest/earliest valid TTO value is included for analysis (i.e., subsequent TTO values of an AE that recurred in the same patient were discarded). For each AE term in the unified dataset, the total count of valid TTO entries was calculated by summing across all SSRIs (e.g., if SSRI-1 reported six nausea events and SSRIs 2-6 reported 10 each, the combined count equals 56). After summation, event terms with fewer than or equal to 10 valid TTO observations were removed. The final dataset comprised 1,428 distinct event terms, and median TTO values for each event term were presented in the Appendices section. This data cleaning process reduced the dataset from the raw ICSR totals to a final analytical sample of 309,775 ICSRs with 1,428 distinct MedDRA Preferred Terms.

Inclusion criteria comprised all ICSRs in VigiBase that listed at least one of the six SSRIs (sertraline, fluoxetine, fluvoxamine, paroxetine, citalopram, or escitalopram) as a suspect drug and contained valid dates for both drug initiation and AE onset.

Exclusion criteria included reports lacking essential temporal information (missing drug start date or event onset date), reports yielding negative or implausible TTO values, duplicate records, and AE terms with ≤10 valid TTO observations after aggregation.

TTO calculation and classification

TTO was defined as the interval from the documented start of SSRI therapy to the onset of the AE, consistent with recent pharmacovigilance practice. Median TTO was used for each event term, given its robustness to skew and outliers in spontaneous reporting data. TTO classification schemes in the literature vary, with many studies evaluating early onset within 30 days and late onset over longer treatment durations. While no single universal cutoff exists, pragmatically classifying AEs as early (median TTO < 28 days) versus late (median TTO > 28 days) aligns with several recent pharmacovigilance analyses that use similar early/late demarcations (e.g., within the first month versus later treatment phases) and acknowledges both clinical treatment cycles and real-world monitoring practices [8]. It approximates one standard prescription cycle, aligns with typical follow-up intervals in antidepressant initiation studies, and represents a pragmatic midpoint between Hu et al.'s [3] 14-day definition of acute AEs and Bull et al.'s [4] 90-day discontinuation window.

Data analysis

All descriptive analyses were conducted at the MedDRA Preferred Term level. For each AE term, the total number of valid TTO observations, median TTO, and interquartile range (IQR) were calculated. Event terms were ranked according to median TTO to explore temporal patterns of SSRI-associated AEs. AEs were categorized as early- or late-onset based on the predefined median TTO threshold of 28 days. Frequencies of early- and late-onset events were summarized descriptively. No stratification by individual SSRI, dose, age, sex, or geographic region was performed, as such variables were incompletely reported in VigiBase and could introduce bias. Given the spontaneous and non-comparative nature of the data, no inferential statistical testing or risk estimation was conducted. Analyses were performed using R statistical software (version 4.3.2; R Foundation for Statistical Computing, Vienna, Austria). Due to the nature of pharmacovigilance data, no inferential comparisons (e.g., adjusted hazard ratios) were conducted; however, patterns of clinical interest were flagged for future hypothesis-driven studies.

Ethical considerations

Access to VigiBase was granted via formal application to the UMC, which governs data access in accordance with WHO pharmacovigilance policies. As the dataset consists entirely of de-identified, spontaneously reported safety data, no ethical approval was required. All analyses were conducted in compliance with WHO and UMC data security protocols and international best practices for pharmacovigilance research.

## Results

A total of 309,775 ICSRs were included in the final dataset after data cleaning. Among these reports, there was a higher proportion of females (208,789, 67.4%) compared to males (96,182, 31.0%), while gender information was missing for 4,804 (1.6%) participants. The age distribution showed that the majority were adults (18-64 years; 125,501, 66.4%), followed by the elderly (≥ 65 years; 52,314, 16.9%). In terms of geographic representation, most case reports were from the European Region (171,805, 55.5%) and the Region of the Americas (94,076, 30.4%) (Table 1).

**Table 1:** Baseline characteristics (n=309,775)

Variable	Description	n (%)
Gender	Female	208,789 (67.41)
	Male	96,182 (31.0)
	Not known	4,804 (1.6)
Age group	≤17 years	17,345 (5.6)
	18–44 years	125,501 (40.5)
	45–64 years	80,167 (25.9)
	≥65 years	52,314 (16.9)
	Unknown	34,448 (11.1)
Region	European Region	171,805 (55.5)
	Region of the Americas	94,076 (30.4)
	Western Pacific Region	31,452 (10.2)
	African Region	830 (0.3)
	Eastern Mediterranean	1,361 (0.4)
	South-East Asian	10,251 (3.3)

**Figure 1:**
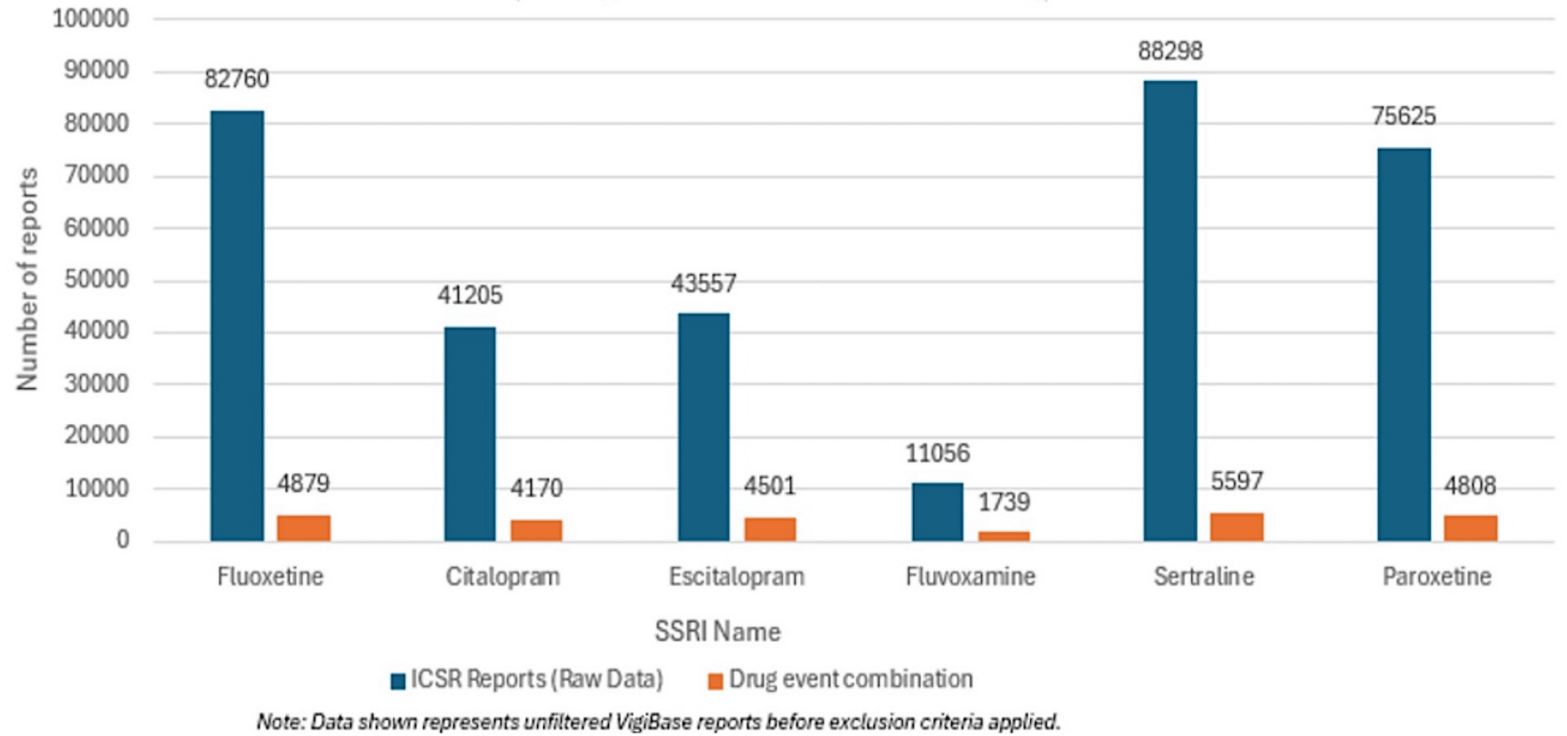
Total number of raw individual case safety reports (ICSRs) and unique drug-event combinations for each SSRI in the VigiBase dataset prior to application of inclusion criteria and data cleaning

Figure 1 and Table 2 present the total number of ICSRs and unique drug-event combinations for each SSRI. Sertraline and fluoxetine had the highest ICSR counts, while fluvoxamine had the lowest. This trend aligns with global prescription patterns: fluoxetine (introduced in 1987) and sertraline (early 1990s) have been widely prescribed worldwide [9], whereas fluvoxamine's use remains indication-specific (primarily for obsessive-compulsive disorder and social anxiety disorder) and less common [10].

**Table 2:** Total number of adverse events reported for each SSRI in the VigiBase dataset SSRI: selective serotonin reuptake inhibitor; ICSR: individual case safety reports

SSRI Name	ICSR Reports (Raw Data)	Drug-Event Combination
Fluoxetine	82760	4879
Citalopram	41205	4170
Escitalopram	43557	4501
Fluvoxamine	11056	1739
Sertraline	88298	5597
Paroxetine	75625	4808

After applying the data cleaning procedures described in the Methods section, the final analytical dataset comprised 309,775 ICSRs with a total of 1,428 MedDRA Preferred Terms. Of these 1428 events, median TTO values ranged from immediate (0 days) to markedly delayed (959.5 days); 515 events were late-onset events (i.e., median TTO > 28 days), while the remaining 913 events were early-onset events (median TTO ≤ 28 days). See the Appendices section for a complete list of these events and their median TTO values.

**Figure 2:**
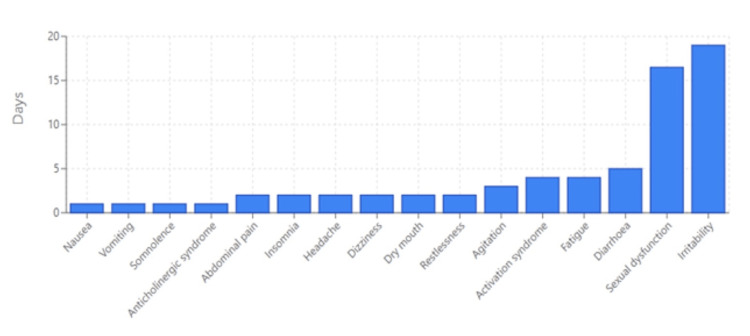
Top early-onset adverse events (median TTO ≤ 28 days)

Early-onset AEs (Figure 2) were predominantly gastrointestinal, neurological, anticholinergic, or related to activation. Examples of such early-onset events include nausea, with a median TTO of just one day, followed by insomnia and headache, both appearing at two days. Agitation and dizziness also emerged early, with median TTOs of three and two days, respectively. Restlessness, anticholinergic syndrome, and dry mouth were similarly observed during the first two days of treatment, whereas fatigue occurred with a median TTO of four days.

**Figure 3:**
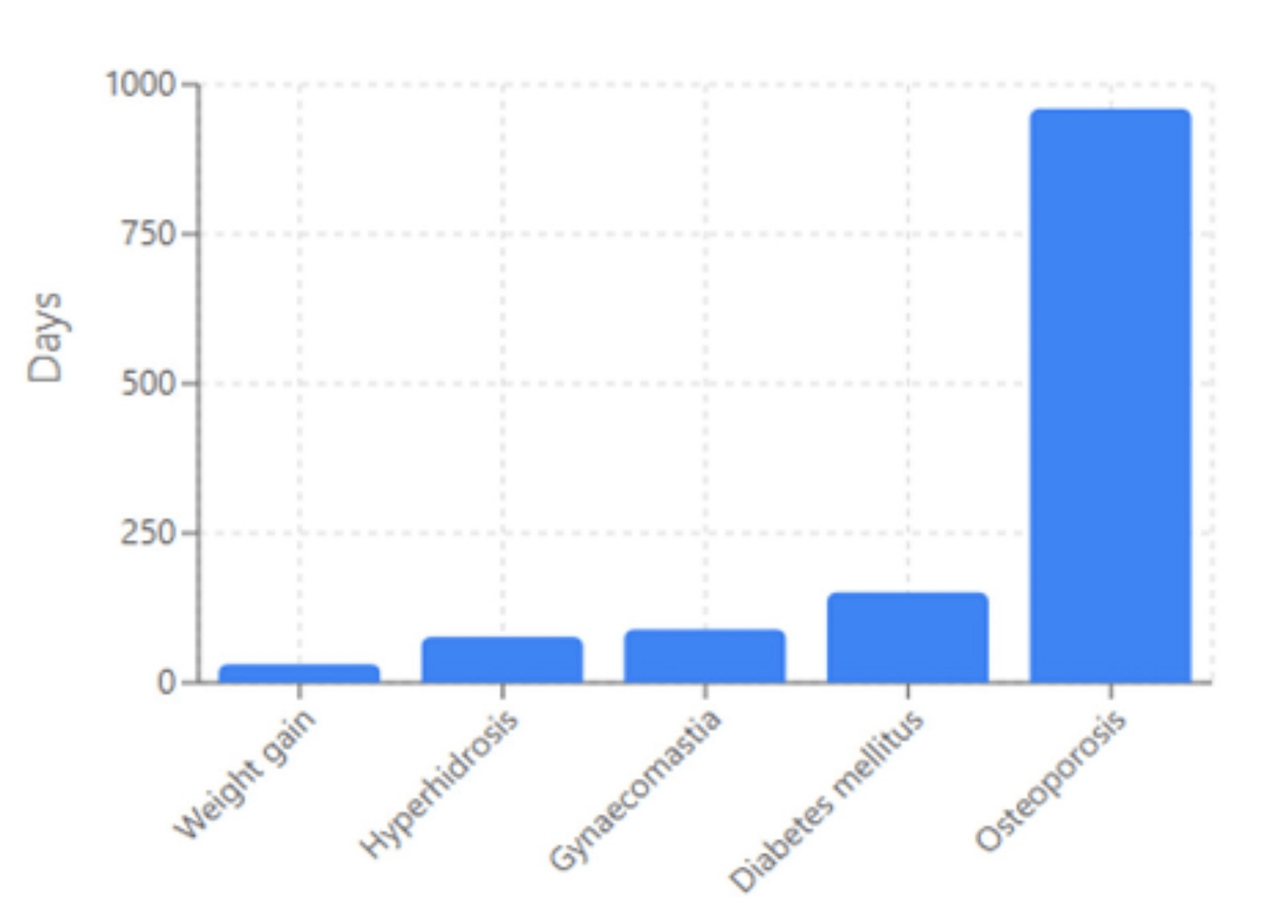
Top late-onset adverse events (median TTO > 28 days)

In contrast, late-onset AEs mainly encompassed metabolic and endocrine complications (Figure 3). Among the most clinically significant late-onset events was weight gain, with a median TTO of 31 days. Hyperhidrosis (increased sweating) appeared later, with a median TTO of 76.5 days, while more severe metabolic disturbances, such as diabetes mellitus, developed after a median of 151 days. Notably, osteoporosis was the latest-onset event, with a median TTO of 959.5 days.

## Discussion

This study demonstrates clear and reproducible temporal patterns in AEs associated with SSRIs, with approximately two-thirds of reported events occurring within the first 28 days of treatment initiation and the remainder emerging later in the treatment course. Early-onset AEs were predominantly gastrointestinal, neurological, anticholinergic, or activation-related, whereas late-onset AEs were largely metabolic or endocrine in nature. This distinct temporal clustering observed in our dataset suggests that SSRI tolerability is dynamic and evolves over time, reflecting underlying pharmacodynamic mechanisms rather than uniform reporting behavior. While similar temporal frameworks have been explored in other antidepressant classes, the consistency and breadth of early-late separation observed here underscore a characteristic pattern associated with SSRIs.

In our analysis, early-onset AEs (median TTO <28 days) were dominated by gastrointestinal, neurological, anticholinergic, and activation-related symptoms, with nausea, insomnia, headache, dizziness, agitation, and restlessness among the most rapidly emerging events. These findings are consistent with clinical observations reported by Strawn et al. [2], who found that such symptoms tend to emerge early in treatment, especially in younger populations, and may subside with continued use. Recent pharmacovigilance studies utilizing the FDA Adverse Event Reporting System have corroborated these patterns, demonstrating that gastrointestinal and neurological symptoms constitute the most frequently reported early-onset AEs in real-world settings [11].

Importantly, sexual dysfunction was also categorized as an early-onset event in our study (median TTO: 16.5 days), corroborating results from Hu et al. [3], where nearly 70% of patients experiencing SSRI-associated sexual dysfunction reported symptom onset within the first two weeks of therapy. This finding aligns with prior literature indicating that sexual side effects may occur early in therapy and, in some contexts (e.g., dapoxetine use for premature ejaculation), are known to emerge acutely. Contemporary research has further highlighted the complexity of SSRI-induced sexual dysfunction, with studies demonstrating that sexual side effects can manifest within days of treatment initiation and may persist even after discontinuation, leading to the recognition of post-SSRI sexual dysfunction (PSSD) as a distinct clinical entity [12,13]. A recent Israeli cohort study estimated that approximately 2.3% of males treated with serotonergic antidepressants may develop persistent erectile dysfunction, emphasizing the clinical significance of early recognition and management [14].

Late-onset AEs in our study (median TTO >28 days) were primarily metabolic and endocrine disturbances, including weight gain, hyperhidrosis, diabetes mellitus, osteoporosis, and gynecomastia. These findings align with those of Strawn et al. [2] and Hu et al. [3], who reported delayed onset of metabolic effects, particularly weight gain, with the majority of cases occurring beyond the initial two-week period. A comprehensive pharmacovigilance analysis focusing specifically on SSRI-associated metabolic complications demonstrated that overweight and glucose/lipid metabolism abnormalities show distinct temporal patterns. While short-term SSRI use is often associated with mild weight loss, prolonged treatment can lead to gradual weight gain in a subset of patients, typically after four to six weeks, and metabolic syndrome developing over months to years of continuous therapy [11]. Notably, osteoporosis emerged as the most delayed AE (median TTO: 959.5 days), underscoring the need for vigilance in long-term SSRI users. This finding is supported by emerging evidence suggesting that chronic SSRI use may interfere with bone metabolism through serotonin receptor-mediated mechanisms, particularly in older adults [15].

The clinical implications of these findings are clinically meaningful. Early-onset AEs, although often transient, may impact adherence if not proactively addressed. Interventions such as gradual dose titration, temporary symptom-targeted adjunctive therapies, and pre-treatment counseling can improve tolerability and persistence [16,17]. Recent clinical guidelines emphasize the importance of structured patient education programs that address expected early-onset symptoms and provide practical management strategies to enhance treatment retention [18]. On the other hand, late-onset AEs, particularly metabolic and endocrine complications, necessitate longitudinal surveillance. Clinical strategies should include periodic monitoring of body mass index (BMI), fasting glucose, lipid profiles, and, where applicable, bone mineral density assessments. A systematic approach to long-term monitoring has been proposed, incorporating risk stratification based on patient demographics, concurrent medications, and baseline metabolic status [6]. Identifying at-risk patients early may help prevent escalation into clinically significant syndromes such as diabetes or osteoporosis.

Strengths of the study

A major strength of this study is the use of a large, globally representative pharmacovigilance dataset encompassing reports from over 130 countries. This breadth captures real-world SSRI use across diverse populations that are typically under-represented in randomized controlled trials. The large sample size enabled identification of robust temporal trends across a wide range of AEs, providing insights into both early- and late-onset toxicity patterns.

Limitations

These findings must be interpreted with caution, given the inherent limitations of spontaneous reporting data. VigiBase data primarily reflect spontaneous reporting patterns rather than true incidence or causal relationships, and the absolute number of reports is minimal compared to the global population receiving SSRI therapy. Several limitations of this study warrant consideration. First, data were derived from spontaneous reporting via VigiBase, which is subject to reporting bias, particularly underreporting of mild, self-limiting, or socially sensitive symptoms (e.g., sexual dysfunction). Recent methodological studies have demonstrated that spontaneous reporting systems capture only an estimated 1-10% of actual AEs, with sexual dysfunction being particularly underreported due to social stigma and patient reluctance to discuss such symptoms [7]. Second, confounding by indication may influence results; the underlying psychiatric conditions themselves (e.g., depression or anxiety) are known to be associated with systemic metabolic alterations, potentially mimicking or amplifying drug-related effects [19]. Third, the dataset lacked information on SSRI dose, duration of therapy, and treatment switching, limiting the ability to assess dose-response effects or pharmacodynamic relationships. Advanced pharmacovigilance methodologies are increasingly incorporating machine learning approaches to address these limitations and improve signal detection accuracy [20]. Fourth, VigiBase reports do not include standardized demographic or baseline clinical data, such as age, sex, comorbidity, or treatment indication. Consequently, subgroup differences in TTO could not be explored. Some AEs, such as gynecomastia or menstrual disorders, are sex-specific, whereas others (e.g., nausea, fatigue) are common to both sexes; treating them equivalently may obscure biological variation in onset timing.

Future research directions

To further clarify SSRI-related AE timing and risk, future studies should incorporate longitudinal cohort designs that prospectively track symptom onset, standardized AE reporting tools to enhance data comparability, and pharmacogenomics analyses to identify individual-level predictors of susceptibility to early- or late-onset toxicity. Recent advances in precision medicine have identified genetic polymorphisms in cytochrome P450 enzymes and serotonin transporter genes that may predict individual variation in SSRI tolerability and AE susceptibility [21,22]. Additionally, the integration of electronic health records with pharmacovigilance databases offers promising opportunities for real-world evidence generation and continuous safety monitoring [23,24]. These approaches would improve the precision of AE risk stratification and support personalized SSRI prescribing strategies.

## Conclusions

This study identifies distinct temporal patterns of AEs associated with SSRIs, with the majority occurring early in treatment and a substantial proportion emerging later. Early-onset AEs were predominantly gastrointestinal, neurological, and activation-related, whereas late-onset events were mainly metabolic and endocrine in nature, including weight gain, diabetes mellitus, and osteoporosis. These findings highlight that SSRI-associated adverse effects evolve over time and differ meaningfully in their onset profiles. Recognizing these temporal patterns has important implications for patient counseling, expectation setting, and long-term treatment planning in individuals receiving SSRI therapy.

## Appendices

**Table 3:** Median time-to-onset for each event

MedDRA PT Name	Median Time-to-Onset	Week
Osteoporosis	959.5	> 4 weeks
Breast cancer	821.5	> 4 weeks
Neutrophil count increased	802.5	> 4 weeks
Mitral valve disease	597.74236	> 4 weeks
Ligament sprain	591.5	> 4 weeks
Breast neoplasm	577	> 4 weeks
Pulmonary malformation	540	> 4 weeks
Blood testosterone decreased	436	> 4 weeks
Rib fracture	425	> 4 weeks
Exomphalos	413	> 4 weeks
Hypoplastic left heart syndrome	380	> 4 weeks
Autism spectrum disorder	367	> 4 weeks
Dementia Alzheimer's type	366	> 4 weeks
Maculopathy	366	> 4 weeks
Lower limb fracture	365.24236	> 4 weeks
Premature rupture of membranes	365.24236	> 4 weeks
Macular degeneration	365	> 4 weeks
Otitis media	365	> 4 weeks
Performance status decreased	365	> 4 weeks
Pulmonary arterial hypertension	364	> 4 weeks
Subdural haemorrhage	355	> 4 weeks
Angina unstable	353	> 4 weeks
Aortic valve incompetence	350	> 4 weeks
Congenital aortic valve incompetence	339	> 4 weeks
Mitral valve prolapse	334.5	> 4 weeks
Plagiocephaly	317	> 4 weeks
Hernia	313.5	> 4 weeks
Fibromyalgia	306	> 4 weeks
Social anxiety disorder	304.25	> 4 weeks
Bicuspid aortic valve	304	> 4 weeks
Hepatic cirrhosis	301.40278	> 4 weeks
Heart disease congenital	299	> 4 weeks
Right atrial dilatation	296	> 4 weeks
Hepatic cancer	293	> 4 weeks
Coronary artery disease	291	> 4 weeks
Normal newborn	289	> 4 weeks
Cyanosis neonatal	286.5	> 4 weeks
Ventricular hypertrophy	284.5	> 4 weeks
Dilatation ventricular	281	> 4 weeks
Pulmonary artery stenosis congenital	277.5	> 4 weeks
Cardiac valve disease	277	> 4 weeks
Caesarean section	275.5	> 4 weeks
Dependence	275	> 4 weeks
Osteonecrosis	273.931945	> 4 weeks
Premature labour	273.5	> 4 weeks
Newborn persistent pulmonary hypertension	272	> 4 weeks
Mitral valve incompetence	271.96597	> 4 weeks
Double outlet right ventricle	271	> 4 weeks
Pulmonary valve stenosis	269	> 4 weeks
Talipes	269	> 4 weeks
Hypertonia neonatal	268	> 4 weeks
Large for dates baby	265	> 4 weeks
Neonatal hypoxia	265	> 4 weeks
Pulmonary valve incompetence	265	> 4 weeks
Somnolence neonatal	265	> 4 weeks
Syndactyly	263.46597	> 4 weeks
Persistent foetal circulation	263	> 4 weeks
Congenital anomaly	262	> 4 weeks
Multiple congenital abnormalities	262	> 4 weeks
Chronic fatigue syndrome	259	> 4 weeks
Lower respiratory tract infection	257.5	> 4 weeks
Congenital aortic stenosis	257	> 4 weeks
Small for dates baby	256.5	> 4 weeks
Cleft palate	255.5	> 4 weeks
Congenital musculoskeletal disorder of limbs	253	> 4 weeks
Tethered oral tissue	252	> 4 weeks
Pericardial effusion	250	> 4 weeks
Pregnancy	250	> 4 weeks
Spina bifida	249.5	> 4 weeks
Cleft lip	248.5	> 4 weeks
Influenza	248	> 4 weeks
Polydactyly	248	> 4 weeks
Ventricular septal defect	246.25	> 4 weeks
Atelectasis	246	> 4 weeks
Coarctation of the aorta	246	> 4 weeks
Hydronephrosis	246	> 4 weeks
Pulmonary valve stenosis congenital	246	> 4 weeks
Drug dependence	245	> 4 weeks
Fallot's tetralogy	245	> 4 weeks
Aortic stenosis	244	> 4 weeks
Drug withdrawal syndrome neonatal	244	> 4 weeks
Left ventricular hypertrophy	244	> 4 weeks
Respiratory disorder neonatal	244	> 4 weeks
Agitation neonatal	243.74757	> 4 weeks
Atrial septal defect	243.49514	> 4 weeks
Cryptorchism	243.49514	> 4 weeks
Hypospadias	243.49514	> 4 weeks
Low birth weight baby	243.49514	> 4 weeks
Neonatal disorder	243.49514	> 4 weeks
Disability	243	> 4 weeks
Pulmonary hypertension	242.75	> 4 weeks
Benign breast neoplasm	242.25	> 4 weeks
Cardiac murmur	242	> 4 weeks
Right ventricular hypertrophy	242	> 4 weeks
Neonatal respiratory distress	241.99757	> 4 weeks
Failure to thrive	241.5	> 4 weeks
Congenital cardiovascular anomaly	240.5	> 4 weeks
Tooth loss	240.25	> 4 weeks
Developmental delay	240	> 4 weeks
Patent ductus arteriosus	240	> 4 weeks
Pneumothorax	240	> 4 weeks
Cardiomegaly	238.5	> 4 weeks
Exposure during pregnancy	238	> 4 weeks
Selective eating disorder	238	> 4 weeks
Tremor neonatal	238	> 4 weeks
Anomalous pulmonary venous connection	237	> 4 weeks
Pulmonary artery stenosis	235.5	> 4 weeks
Hypoglycaemia neonatal	234.24757	> 4 weeks
Neonatal respiratory distress syndrome	231	> 4 weeks
Pulmonary hypoplasia	231	> 4 weeks
Cytogenetic abnormality	230.25	> 4 weeks
Cleft lip and palate	230	> 4 weeks
Ventricular hypoplasia	230	> 4 weeks
Apgar score low	229.74757	> 4 weeks
Cyst	229.25	> 4 weeks
Coeliac disease	229	> 4 weeks
Hydrocephalus	228.5	> 4 weeks
Bradycardia neonatal	228.276735	> 4 weeks
Cardiac failure congestive	228	> 4 weeks
Surgery	227.5	> 4 weeks
Neonatal seizure	223	> 4 weeks
Transposition of the great vessels	221	> 4 weeks
Dysmorphism	218.75	> 4 weeks
Microcephaly	218.25	> 4 weeks
Tricuspid valve incompetence	218	> 4 weeks
Craniosynostosis	216	> 4 weeks
Infantile apnoea	216	> 4 weeks
Premature delivery	216	> 4 weeks
Dental caries	214.5	> 4 weeks
Uterine leiomyoma	214	> 4 weeks
Haemangioma congenital	213.05833	> 4 weeks
Cataract	213	> 4 weeks
Aortic valve disease	212	> 4 weeks
Hypotonia neonatal	212	> 4 weeks
Speech disorder developmental	207	> 4 weeks
Foot deformity	205.5	> 4 weeks
Electric shock	200	> 4 weeks
Neoplasm malignant	198.5	> 4 weeks
Bronchiolitis	198	> 4 weeks
Neonatal respiratory depression	193	> 4 weeks
Abortion spontaneous	191	> 4 weeks
Metabolic disorder	191	> 4 weeks
Subdural haematoma	189.25	> 4 weeks
Premature baby	189	> 4 weeks
Product complaint	189	> 4 weeks
Aorta hypoplasia	187	> 4 weeks
Drug withdrawal syndrome	186	> 4 weeks
Cardiovascular disorder	183	> 4 weeks
Thermal burn	183	> 4 weeks
Pleurisy	182.5	> 4 weeks
Growth retardation	179.310765	> 4 weeks
Cerebral haematoma	178	> 4 weeks
Cerebral disorder	177	> 4 weeks
Live birth	177	> 4 weeks
Lung disorder	173.5	> 4 weeks
Temperature regulation disorder	173.5	> 4 weeks
Haemorrhage intracranial	171.25	> 4 weeks
Foetal growth restriction	170	> 4 weeks
Hypertriglyceridaemia	170	> 4 weeks
Blood cholesterol increased	166.75	> 4 weeks
Splenomegaly	166.5	> 4 weeks
Hepatic steatosis	164	> 4 weeks
Neoplasm	163	> 4 weeks
Unintended pregnancy	162	> 4 weeks
Withdrawal syndrome	161.25	> 4 weeks
Renal disorder	161	> 4 weeks
Interstitial lung disease	159.59236	> 4 weeks
Abortion induced	159	> 4 weeks
Cardiomyopathy	157.5	> 4 weeks
Malabsorption	155	> 4 weeks
Cardiac disorder	153	> 4 weeks
Lower gastrointestinal haemorrhage	152.18472	> 4 weeks
Systemic lupus erythematosus	152	> 4 weeks
Diabetes mellitus	151	> 4 weeks
Ear infection	150	> 4 weeks
Type 2 diabetes mellitus	149	> 4 weeks
Ischaemic stroke	148	> 4 weeks
Drug withdrawal headache	147.5	> 4 weeks
Sleep terror	147	> 4 weeks
Pre-eclampsia	146.5	> 4 weeks
Sjogren's syndrome	146.49757	> 4 weeks
Atrial tachycardia	146	> 4 weeks
Rheumatoid arthritis	145.75	> 4 weeks
Chronic obstructive pulmonary disease	145	> 4 weeks
Retinal haemorrhage	144.5	> 4 weeks
High-density lipoprotein decreased	144	> 4 weeks
Foetal distress syndrome	142.5	> 4 weeks
Abortion missed	141	> 4 weeks
Anencephaly	141	> 4 weeks
Foetal death	141	> 4 weeks
Hypocalcaemia	141	> 4 weeks
Polyneuropathy	139.25	> 4 weeks
Pregnancy on oral contraceptive	137.37396	> 4 weeks
Abortion	137	> 4 weeks
Parkinson's disease	134	> 4 weeks
Dysgraphia	133.5	> 4 weeks
Haemarthrosis	133	> 4 weeks
Cerebral infarction	132	> 4 weeks
Right ventricular failure	132	> 4 weeks
Maternal exposure during pregnancy	131.75	> 4 weeks
Thyroxine decreased	131	> 4 weeks
Glucose tolerance impaired	130.75	> 4 weeks
Ejection fraction abnormal	126.5	> 4 weeks
Thrombosis	125	> 4 weeks
Optic ischaemic neuropathy	123.25	> 4 weeks
Cellulitis	123	> 4 weeks
Frustration tolerance decreased	123	> 4 weeks
Intellectual disability	123	> 4 weeks
Lung infiltration	122.5	> 4 weeks
Staphylococcal infection	122.5	> 4 weeks
Cerebral haemorrhage	122	> 4 weeks
Nephrolithiasis	122	> 4 weeks
Stillbirth	122	> 4 weeks
Intestinal obstruction	121	> 4 weeks
Angiopathy	120.5	> 4 weeks
Femur fracture	120	> 4 weeks
Hyperlipidaemia	120	> 4 weeks
Electric shock sensation	119	> 4 weeks
Varicose vein	119	> 4 weeks
Optic neuritis	117	> 4 weeks
Vocal cord paralysis	116.5	> 4 weeks
Pulmonary fibrosis	116	> 4 weeks
Aortic valve stenosis	114	> 4 weeks
Intracranial pressure increased	114	> 4 weeks
Compulsive shopping	113	> 4 weeks
Intraventricular haemorrhage	111.155555	> 4 weeks
Hypersensitivity pneumonitis	109	> 4 weeks
Deep vein thrombosis	108.5	> 4 weeks
COVID-19	106.25	> 4 weeks
Blood thyroid-stimulating hormone increased	105	> 4 weeks
Rebound effect	105	> 4 weeks
Bundle branch block right	104.5	> 4 weeks
Alveolitis	102	> 4 weeks
Ovarian cyst	102	> 4 weeks
Pleural effusion	102	> 4 weeks
Jaundice neonatal	100	> 4 weeks
Drug screen false positive	98	> 4 weeks
Rosacea	98	> 4 weeks
Hyponatraemic syndrome	97	> 4 weeks
Bacterial infection	95	> 4 weeks
Papilloedema	95	> 4 weeks
Hypercholesterolaemia	93	> 4 weeks
Loss of employment	92	> 4 weeks
Bronchitis	91.5	> 4 weeks
Musculoskeletal discomfort	91	> 4 weeks
Cholelithiasis	90.5	> 4 weeks
Blindness unilateral	90	> 4 weeks
Gynaecomastia	89	> 4 weeks
Thyroiditis	87.655555	> 4 weeks
Breast discomfort	87	> 4 weeks
Road traffic accident	86.655555	> 4 weeks
Retinal disorder	86	> 4 weeks
Sensory disturbance	86	> 4 weeks
Drug screen positive	85	> 4 weeks
Duodenal ulcer perforation	85	> 4 weeks
Hair growth abnormal	84	> 4 weeks
Phlebitis	83.25	> 4 weeks
Hypovolaemia	81	> 4 weeks
Iron deficiency anaemia	80.5	> 4 weeks
Myocardial ischaemia	80	> 4 weeks
Skin ulcer	80	> 4 weeks
Product dose omission issue	79	> 4 weeks
Trigeminal neuralgia	78.5	> 4 weeks
Goitre	77	> 4 weeks
Haematocrit decreased	77	> 4 weeks
Hypoxia	77	> 4 weeks
Oesophageal ulcer	76.75	> 4 weeks
Hyperprolactinaemia	76.5	> 4 weeks
Respiratory distress	76	> 4 weeks
Vitiligo	76	> 4 weeks
Muscle atrophy	75	> 4 weeks
Pulmonary congestion	75	> 4 weeks
Embolism	73	> 4 weeks
Nephrotic syndrome	73	> 4 weeks
Aplastic anaemia	72.5	> 4 weeks
Cerebral ischaemia	72.5	> 4 weeks
Diabetic ketoacidosis	72.5	> 4 weeks
Hyperthyroidism	72.5	> 4 weeks
Hiatus hernia	72.25	> 4 weeks
Tubulointerstitial nephritis	72	> 4 weeks
Yellow skin	71	> 4 weeks
Blood thyroid-stimulating hormone decreased	70.5	> 4 weeks
Hypothyroidism	70	> 4 weeks
Left ventricular failure	70	> 4 weeks
Pulmonary oedema	69.5	> 4 weeks
Carpal tunnel syndrome	69	> 4 weeks
Bleeding time prolonged	68	> 4 weeks
Neonatal asphyxia	67.5	> 4 weeks
Mucosal dryness	67	> 4 weeks
Upper respiratory tract infection	66	> 4 weeks
Temperature intolerance	64.5	> 4 weeks
Haemorrhagic stroke	64	> 4 weeks
Joint dislocation	63	> 4 weeks
Skin striae	63	> 4 weeks
Oligomenorrhoea	62.5	> 4 weeks
Alcohol intolerance	62	> 4 weeks
Exposure via breast milk	62	> 4 weeks
Bursitis	61	> 4 weeks
Cerebrovascular disorder	61	> 4 weeks
Food interaction	61	> 4 weeks
Hepatomegaly	61	> 4 weeks
Hyperuricaemia	61	> 4 weeks
Lichen planus	61	> 4 weeks
Pericarditis	61	> 4 weeks
Tendonitis	60.937155	> 4 weeks
Atrioventricular block first degree	60.87431	> 4 weeks
Colitis microscopic	60.87431	> 4 weeks
Muscle fatigue	60.87431	> 4 weeks
Rabbit syndrome	60.87431	> 4 weeks
White blood cell count increased	60.87431	> 4 weeks
Thyroid disorder	60.75	> 4 weeks
Therapy non-responder	60	> 4 weeks
Treatment noncompliance	59.5	> 4 weeks
Bipolar I disorder	59	> 4 weeks
Pneumonitis	59	> 4 weeks
Gallbladder disorder	58.5	> 4 weeks
Homicidal ideation	58.5	> 4 weeks
Breast enlargement	57.5	> 4 weeks
Thrombocytopenic purpura	56.5	> 4 weeks
Breast mass	56	> 4 weeks
Homicide	56	> 4 weeks
Subarachnoid haemorrhage	56	> 4 weeks
Upper gastrointestinal haemorrhage	55.25	> 4 weeks
Craniocerebral injury	55.21875	> 4 weeks
Hypertrichosis	55	> 4 weeks
Renal colic	55	> 4 weeks
Sunburn	55	> 4 weeks
Hypoacusis	54	> 4 weeks
Ocular hypertension	53.655555	> 4 weeks
Asthma	53	> 4 weeks
Pigmentation disorder	53	> 4 weeks
Ulcerative keratitis	52.75	> 4 weeks
Oxygen saturation decreased	52	> 4 weeks
Partial seizures	52	> 4 weeks
Physical assault	52	> 4 weeks
Obesity	51.75	> 4 weeks
Blindness	51.5	> 4 weeks
Premature ejaculation	51	> 4 weeks
Respiratory failure	51	> 4 weeks
Galactorrhoea	50	> 4 weeks
Duodenitis	49	> 4 weeks
Herpes zoster	49	> 4 weeks
Lichenoid keratosis	49	> 4 weeks
Synovitis	49	> 4 weeks
Increased tendency to bruise	48.5	> 4 weeks
Drug tolerance increased	47.5	> 4 weeks
Impatience	47	> 4 weeks
Pneumonia	47	> 4 weeks
Injury	46.5	> 4 weeks
Nasopharyngitis	46	> 4 weeks
Pulmonary haemorrhage	46	> 4 weeks
Weight gain poor	46	> 4 weeks
Colitis	45.5	> 4 weeks
Foetal exposure during pregnancy	45.25	> 4 weeks
Hypersexuality	45	> 4 weeks
Neuropathy peripheral	45	> 4 weeks
Limb injury	44.5	> 4 weeks
Infertility male	44.25	> 4 weeks
Breast tenderness	44	> 4 weeks
Cardiac failure	44	> 4 weeks
Cyanosis	44	> 4 weeks
Gastric ulcer	44	> 4 weeks
Haemolytic anaemia	44	> 4 weeks
Sepsis	44	> 4 weeks
Duodenal ulcer	43.5	> 4 weeks
Blindness transient	43	> 4 weeks
Brain injury	43	> 4 weeks
Crohn's disease	43	> 4 weeks
Intraocular pressure increased	43	> 4 weeks
Endocrine disorder	42	> 4 weeks
Hypochromic anaemia	42	> 4 weeks
Pulmonary embolism	42	> 4 weeks
Vitamin D decreased	41.5	> 4 weeks
Acute myocardial infarction	41	> 4 weeks
Bundle branch block	41	> 4 weeks
Ileus	41	> 4 weeks
Myocardial infarction	41	> 4 weeks
Tardive dyskinesia	41	> 4 weeks
Agoraphobia	40.5	> 4 weeks
Accident	39.5	> 4 weeks
Antipsychotic drug level increased	39	> 4 weeks
Fracture	39	> 4 weeks
Multiple sclerosis	39	> 4 weeks
Tearfulness	39	> 4 weeks
Viral infection	39	> 4 weeks
Proteinuria	38.75	> 4 weeks
Antinuclear antibody positive	38	> 4 weeks
Hepatic necrosis	38	> 4 weeks
Onychoclasis	37.71875	> 4 weeks
Pancreatitis	37.5	> 4 weeks
Brain oedema	37	> 4 weeks
Feeling of despair	37	> 4 weeks
Hepatic enzyme abnormal	37	> 4 weeks
Hypomenorrhoea	37	> 4 weeks
Motion sickness	37	> 4 weeks
Alcoholism	36.5	> 4 weeks
Blood alcohol increased	36.5	> 4 weeks
Dyslipidaemia	36.5	> 4 weeks
Alcohol interaction	36	> 4 weeks
Alopecia areata	36	> 4 weeks
Ascites	36	> 4 weeks
Hair colour changes	36	> 4 weeks
Nervous system disorder	36	> 4 weeks
Pancreatitis acute	36	> 4 weeks
Postmenopausal haemorrhage	36	> 4 weeks
Skin depigmentation	36	> 4 weeks
Tooth discolouration	36	> 4 weeks
Apnoea	35.5	> 4 weeks
Dyspnoea exertional	35.5	> 4 weeks
Adverse event	35	> 4 weeks
Anaemia	35	> 4 weeks
Breast discharge	35	> 4 weeks
Head injury	35	> 4 weeks
Hypotrichosis	35	> 4 weeks
Myocarditis	35	> 4 weeks
Red blood cell count decreased	35	> 4 weeks
Vulvovaginal dryness	35	> 4 weeks
Bladder disorder	34.5	> 4 weeks
Hemiparesis	34.5	> 4 weeks
Torsade de pointes	34.5	> 4 weeks
Albuminuria	34	> 4 weeks
Anger	34	> 4 weeks
Hyperacusis	34	> 4 weeks
Spontaneous haematoma	34	> 4 weeks
Testicular disorder	34	> 4 weeks
Visual field defect	34	> 4 weeks
Colitis ulcerative	33.5	> 4 weeks
Hyperbilirubinaemia	33.5	> 4 weeks
Retinal vein thrombosis	33.25	> 4 weeks
Amenorrhoea	33	> 4 weeks
Immune system disorder	33	> 4 weeks
Retinal detachment	33	> 4 weeks
Social avoidant behaviour	32.5	> 4 weeks
Alopecia	32	> 4 weeks
Blood triglycerides increased	32	> 4 weeks
Electroencephalogram abnormal	32	> 4 weeks
Judgement impaired	32	> 4 weeks
Lymphopenia	32	> 4 weeks
Red blood cell sedimentation rate increased	32	> 4 weeks
Ventricular arrhythmia	32	> 4 weeks
Seborrhoea	31.75	> 4 weeks
Granulocytopenia	31.5	> 4 weeks
Oral candidiasis	31.25	> 4 weeks
Amylase increased	31	> 4 weeks
Bipolar disorder	31	> 4 weeks
Cerebrovascular accident	31	> 4 weeks
Deafness	31	> 4 weeks
Drug-induced liver injury	31	> 4 weeks
Eye haemorrhage	31	> 4 weeks
Hepatic encephalopathy	31	> 4 weeks
Hepatorenal syndrome	31	> 4 weeks
Infection	31	> 4 weeks
Inflammation	31	> 4 weeks
Muscle haemorrhage	31	> 4 weeks
Myelosuppression	31	> 4 weeks
Nail disorder	31	> 4 weeks
Pemphigoid	31	> 4 weeks
Prostatic-specific antigen increased	31	> 4 weeks
Urine analysis abnormal	31	> 4 weeks
Visual acuity reduced	31	> 4 weeks
Weight increased	31	> 4 weeks
Gastric ulcer haemorrhage	30.71875	> 4 weeks
Hair texture abnormal	30.5	> 4 weeks
Lipase increased	30.5	> 4 weeks
Motor dysfunction	30.5	> 4 weeks
Nasal dryness	30.5	> 4 weeks
Neuritis	30.5	> 4 weeks
Premenstrual syndrome	30.5	> 4 weeks
Sleep apnoea syndrome	30.5	> 4 weeks
Emotional poverty	30.46875	> 4 weeks
Abnormal sensation in the eye	30.4375	> 4 weeks
Abscess	30.4375	> 4 weeks
Death	30.4375	> 4 weeks
Essential tremor	30.4375	> 4 weeks
Hypertransaminasaemia	30.4375	> 4 weeks
Impaired healing	30.4375	> 4 weeks
Laryngitis	30.4375	> 4 weeks
Photosensitivity reaction	30.4375	> 4 weeks
Platelet count decreased	30.4375	> 4 weeks
Skin hyperpigmentation	30.4375	> 4 weeks
Glaucoma	30.25	> 4 weeks
Hepatitis cholestatic	30.21875	> 4 weeks
Hospitalisation	30.21875	> 4 weeks
Rectal haemorrhage	30.21875	> 4 weeks
Alcohol problem	30	> 4 weeks
Aspartate aminotransferase	30	> 4 weeks
Bulimia nervosa	30	> 4 weeks
Emotional disorder	30	> 4 weeks
Erythema nodosum	30	> 4 weeks
Hostility	30	> 4 weeks
Influenza-like illness	30	> 4 weeks
Memory impairment	30	> 4 weeks
Thrombocytosis	30	> 4 weeks
Menstruation delayed	29.71875	> 4 weeks
Amnesia	29.25	> 4 weeks
Overweight	29.21875	> 4 weeks
Arthritis	29	> 4 weeks
Blood urea increased	29	> 4 weeks
Breast pain	29	> 4 weeks
Eosinophilic pneumonia	29	> 4 weeks
Gingival pain	29	> 4 weeks
Hypersensitivity vasculitis	29	> 4 weeks
Hypophagia	29	> 4 weeks
Iritis	29	> 4 weeks
Major depression	29	> 4 weeks
Myositis	29	> 4 weeks
Reversible cerebral vasoconstriction syndrome	29	> 4 weeks
Skin discolouration	29	> 4 weeks
Arthropathy	28.75	> 4 weeks
Salivary gland enlargement	28.75	> 4 weeks
Blood alkaline phosphatase increased	28.5	> 4 weeks
Bone marrow failure	28.5	> 4 weeks
Ill-defined disorder	28.5	> 4 weeks
Mastitis	28.5	> 4 weeks
Venous thrombosis	28.5	> 4 weeks
Blood uric acid increased	28	<= 4 weeks
Crying	28	<= 4 weeks
Ecchymosis	28	<= 4 weeks
Gastrointestinal haemorrhage	28	<= 4 weeks
Haematuria	28	<= 4 weeks
Haemoptysis	28	<= 4 weeks
Haemorrhage	28	<= 4 weeks
Hepatitis toxic	28	<= 4 weeks
Myopia	28	<= 4 weeks
Orgasmic sensation decreased	28	<= 4 weeks
Psychiatric symptom	28	<= 4 weeks
Anhedonia	27.5	<= 4 weeks
Blood glucose increased	27.5	<= 4 weeks
Pathological fracture	27.5	<= 4 weeks
Blood bilirubin increased	27	<= 4 weeks
Choking	27	<= 4 weeks
Coagulopathy	27	<= 4 weeks
Contusion	27	<= 4 weeks
Gamma-glutamyltransferase increased	27	<= 4 weeks
Hepatitis	27	<= 4 weeks
Schizophrenia	27	<= 4 weeks
Tooth disorder	27	<= 4 weeks
Duodenal ulcer haemorrhage	26.5	<= 4 weeks
Haemorrhoids	26.5	<= 4 weeks
Lymphocytosis	26.5	<= 4 weeks
Mood swings	26.5	<= 4 weeks
Blood lactate dehydrogenase increased	26	<= 4 weeks
Breast engorgement	26	<= 4 weeks
Liver disorder	26	<= 4 weeks
Purpura	26	<= 4 weeks
Septic shock	26	<= 4 weeks
Skin abrasion	26	<= 4 weeks
Sudden death	26	<= 4 weeks
Tension headache	26	<= 4 weeks
Aphonia	25.5	<= 4 weeks
Blood loss anaemia	25.5	<= 4 weeks
Osteoarthritis	25.5	<= 4 weeks
Energy increased	25.25	<= 4 weeks
Alanine aminotransferase increased	25	<= 4 weeks
Haemoglobin decreased	25	<= 4 weeks
Hypercalcaemia	25	<= 4 weeks
Priapism	25	<= 4 weeks
Conjunctival haemorrhage	24.5	<= 4 weeks
Dysmenorrhoea	24.5	<= 4 weeks
Hepatic failure	24.5	<= 4 weeks
Hypohidrosis	24.5	<= 4 weeks
Pancytopenia	24.5	<= 4 weeks
Haematoma	24	<= 4 weeks
Hypertonic bladder	24	<= 4 weeks
Impaired driving ability	24	<= 4 weeks
Jaundice	24	<= 4 weeks
Leukocytosis	24	<= 4 weeks
Liver function test abnormal	24	<= 4 weeks
Menstrual disorder	24	<= 4 weeks
Food aversion	23.5	<= 4 weeks
Muscle disorder	23.5	<= 4 weeks
Vasculitis	23.5	<= 4 weeks
Ventricular extrasystoles	23.5	<= 4 weeks
Cough	23	<= 4 weeks
Drug level decreased	23	<= 4 weeks
Eyelid ptosis	23	<= 4 weeks
Haematemesis	23	<= 4 weeks
Joint swelling	23	<= 4 weeks
Language disorder	23	<= 4 weeks
Mixed anxiety and depressive disorder	23	<= 4 weeks
Oral herpes	23	<= 4 weeks
Concussion	22.5	<= 4 weeks
Drug level increased	22.5	<= 4 weeks
Hyperglycaemia	22.5	<= 4 weeks
Hypogeusia	22.5	<= 4 weeks
Psoriasis	22.5	<= 4 weeks
Mental impairment	22.35	<= 4 weeks
Abnormal weight gain	22	<= 4 weeks
Aspartate aminotransferase increased	22	<= 4 weeks
Blood albumin decreased	22	<= 4 weeks
Conduction disorder	22	<= 4 weeks
Hepatic enzyme increased	22	<= 4 weeks
Hepatotoxicity	22	<= 4 weeks
Leukopenia	22	<= 4 weeks
Oral mucosal blistering	22	<= 4 weeks
Renal failure	22	<= 4 weeks
Renal impairment	22	<= 4 weeks
Serum sickness	22	<= 4 weeks
Sinusitis	22	<= 4 weeks
Therapy cessation	22	<= 4 weeks
Thrombocytopenia	22	<= 4 weeks
Unevaluable event	22	<= 4 weeks
Encephalopathy	21.75	<= 4 weeks
Hirsutism	21.5	<= 4 weeks
Pneumonitis aspiration	21.5	<= 4 weeks
Menstruation irregular	21.25	<= 4 weeks
Arthralgia	21	<= 4 weeks
Choreoathetosis	21	<= 4 weeks
Drooling	21	<= 4 weeks
Dry skin	21	<= 4 weeks
Haematospermia	21	<= 4 weeks
Hypocoagulable state	21	<= 4 weeks
Male orgasmic disorder	21	<= 4 weeks
Mechanical urticaria	21	<= 4 weeks
Neutrophil count decreased	21	<= 4 weeks
Night sweats	21	<= 4 weeks
Platelet disorder	21	<= 4 weeks
Prothrombin level increased	21	<= 4 weeks
Sneezing	21	<= 4 weeks
Subcutaneous haematoma	21	<= 4 weeks
Abdominal wall haematoma	20.5	<= 4 weeks
Asphyxia	20.5	<= 4 weeks
Eosinophilia	20.5	<= 4 weeks
Myopathy	20.5	<= 4 weeks
Screaming	20.5	<= 4 weeks
Drug withdrawal convulsions	20.21875	<= 4 weeks
Atrioventricular block complete	20	<= 4 weeks
Atrioventricular block second degree	20	<= 4 weeks
Blood creatine phosphokinase increased	20	<= 4 weeks
Diffuse alopecia	20	<= 4 weeks
Erythema multiforme	20	<= 4 weeks
Furuncle	20	<= 4 weeks
Hair disorder	20	<= 4 weeks
Hepatitis acute	20	<= 4 weeks
Liver injury	20	<= 4 weeks
Mania	20	<= 4 weeks
Melaena	20	<= 4 weeks
Tachypnoea	20	<= 4 weeks
Urinary tract infection	20	<= 4 weeks
Wound	20	<= 4 weeks
Haemorrhage subcutaneous	19.75	<= 4 weeks
Lymphadenopathy	19.75	<= 4 weeks
Drop attacks	19.5	<= 4 weeks
Therapeutic product effect incomplete	19.5	<= 4 weeks
Aggression	19	<= 4 weeks
Blood creatinine increased	19	<= 4 weeks
Breast swelling	19	<= 4 weeks
Cognitive disorder	19	<= 4 weeks
Electrocardiogram abnormal	19	<= 4 weeks
Faecaloma	19	<= 4 weeks
Hemiplegia	19	<= 4 weeks
Irritability	19	<= 4 weeks
Jaundice cholestatic	19	<= 4 weeks
Multiple organ dysfunction syndrome	19	<= 4 weeks
Personality disorder	19	<= 4 weeks
Pulse abnormal	19	<= 4 weeks
Skin exfoliation	19	<= 4 weeks
Supraventricular tachycardia	19	<= 4 weeks
Tongue discolouration	19	<= 4 weeks
Torticollis	19	<= 4 weeks
Affective disorder	18.5	<= 4 weeks
Dehydration	18.5	<= 4 weeks
Long QT syndrome	18.5	<= 4 weeks
Stridor	18.5	<= 4 weeks
Violence-related symptom	18.25	<= 4 weeks
Aspiration	18	<= 4 weeks
Cholecystitis	18	<= 4 weeks
Completed suicide	18	<= 4 weeks
Depressive symptom	18	<= 4 weeks
Enuresis	18	<= 4 weeks
Gingivitis	18	<= 4 weeks
Hepatocellular injury	18	<= 4 weeks
Hyperammonaemia	18	<= 4 weeks
Indifference	18	<= 4 weeks
Intention tremor	18	<= 4 weeks
Meningitis	18	<= 4 weeks
Odynophagia	18	<= 4 weeks
Parkinsonism	18	<= 4 weeks
Persecutory delusion	18	<= 4 weeks
Petechiae	18	<= 4 weeks
Sinus headache	18	<= 4 weeks
Transaminases increased	18	<= 4 weeks
Blepharospasm	17.5	<= 4 weeks
Skin lesion	17.5	<= 4 weeks
Vascular purpura	17.5	<= 4 weeks
Product physical issue	17.25	<= 4 weeks
Azotaemia	17	<= 4 weeks
Cardiac arrest	17	<= 4 weeks
Dermatitis bullous	17	<= 4 weeks
Disease recurrence	17	<= 4 weeks
Fixed eruption	17	<= 4 weeks
Food craving	17	<= 4 weeks
Hepatic function abnormal	17	<= 4 weeks
Hypomania	17	<= 4 weeks
Inappropriate affect	17	<= 4 weeks
Incontinence	17	<= 4 weeks
Obsessive-compulsive disorder	17	<= 4 weeks
Oesophagitis	17	<= 4 weeks
Personality change	17	<= 4 weeks
Skin haemorrhage	17	<= 4 weeks
Ventricular tachycardia	17	<= 4 weeks
Drug reaction with eosinophilia and systemic symptoms	16.5	<= 4 weeks
Fungal infection	16.5	<= 4 weeks
Generalised oedema	16.5	<= 4 weeks
Sexual dysfunction	16.5	<= 4 weeks
Strabismus	16.5	<= 4 weeks
Oedema peripheral	16.25	<= 4 weeks
Aphasia	16	<= 4 weeks
Cachexia	16	<= 4 weeks
Delusion	16	<= 4 weeks
Dermatitis contact	16	<= 4 weeks
Epilepsy	16	<= 4 weeks
Flashback	16	<= 4 weeks
Generalised tonic-clonic seizure	16	<= 4 weeks
Gravitational oedema	16	<= 4 weeks
Hypernatraemia	16	<= 4 weeks
Immune thrombocytopenia	16	<= 4 weeks
Loss of libido	16	<= 4 weeks
Polymenorrhoea	16	<= 4 weeks
Thrombophlebitis	16	<= 4 weeks
White blood cell count decreased	16	<= 4 weeks
Gastric haemorrhage	15.5	<= 4 weeks
Migraine with aura	15.5	<= 4 weeks
Obstructive airways disorder	15.5	<= 4 weeks
Rash pustular	15.5	<= 4 weeks
Urinary incontinence	15.5	<= 4 weeks
Renal pain	15.25	<= 4 weeks
Abnormal behaviour	15	<= 4 weeks
Acute hepatic failure	15	<= 4 weeks
Affect lability	15	<= 4 weeks
Agranulocytosis	15	<= 4 weeks
Decreased activity	15	<= 4 weeks
Fall	15	<= 4 weeks
Feelings of worthlessness	15	<= 4 weeks
Menometrorrhagia	15	<= 4 weeks
Neutropenia	15	<= 4 weeks
Raynaud's phenomenon	15	<= 4 weeks
Seizure	15	<= 4 weeks
Skin disorder	15	<= 4 weeks
Skin laceration	15	<= 4 weeks
Transient ischaemic attack	15	<= 4 weeks
Aphthous ulcer	14.5	<= 4 weeks
Conjunctivitis	14.5	<= 4 weeks
Eczema	14.5	<= 4 weeks
Groin pain	14.5	<= 4 weeks
Psychotic disorder	14.5	<= 4 weeks
Schizophreniform disorder	14.5	<= 4 weeks
Skin odour abnormal	14.5	<= 4 weeks
Vitreous floaters	14.5	<= 4 weeks
Activated protein C resistance	14	<= 4 weeks
Acute respiratory distress syndrome	14	<= 4 weeks
Angina pectoris	14	<= 4 weeks
Atrial fibrillation	14	<= 4 weeks
Atrioventricular block	14	<= 4 weeks
Blood potassium decreased	14	<= 4 weeks
Blood urine present	14	<= 4 weeks
Bundle branch block left	14	<= 4 weeks
Catatonia	14	<= 4 weeks
Colour blindness	14	<= 4 weeks
Depression	14	<= 4 weeks
Depression suicidal	14	<= 4 weeks
Dermatitis psoriasiform	14	<= 4 weeks
Diarrhoea haemorrhagic	14	<= 4 weeks
Disturbance in sexual arousal	14	<= 4 weeks
Dry throat	14	<= 4 weeks
Ejaculation delayed	14	<= 4 weeks
Faeces discoloured	14	<= 4 weeks
Flat affect	14	<= 4 weeks
Fluid retention	14	<= 4 weeks
Folliculitis	14	<= 4 weeks
Heavy menstrual bleeding	14	<= 4 weeks
Hepatitis fulminant	14	<= 4 weeks
Hyperaesthesia	14	<= 4 weeks
Hyperkalaemia	14	<= 4 weeks
Hypovolaemic shock	14	<= 4 weeks
Impulse-control disorder	14	<= 4 weeks
Impulsive behaviour	14	<= 4 weeks
Intermenstrual bleeding	14	<= 4 weeks
Libido decreased	14	<= 4 weeks
Libido disorder	14	<= 4 weeks
Mental disorder	14	<= 4 weeks
Mixed liver injury	14	<= 4 weeks
Mood altered	14	<= 4 weeks
Obsessive thoughts	14	<= 4 weeks
Oesophageal spasm	14	<= 4 weeks
Orgasm abnormal	14	<= 4 weeks
Petit mal epilepsy	14	<= 4 weeks
Post-traumatic stress disorder	14	<= 4 weeks
Respiratory disorder	14	<= 4 weeks
Rhinitis	14	<= 4 weeks
Skin irritation	14	<= 4 weeks
Somnambulism	14	<= 4 weeks
Stress	14	<= 4 weeks
Supraventricular extrasystoles	14	<= 4 weeks
Ventricular fibrillation	14	<= 4 weeks
Weight decreased	14	<= 4 weeks
Acne	13.5	<= 4 weeks
Coagulation time prolonged	13.5	<= 4 weeks
Hypomagnesaemia	13.5	<= 4 weeks
Dementia	13.25	<= 4 weeks
Anal haemorrhage	13	<= 4 weeks
Blood prolactin increased	13	<= 4 weeks
Cholestatic liver injury	13	<= 4 weeks
Cutaneous vasculitis	13	<= 4 weeks
Eye disorder	13	<= 4 weeks
Gingival bleeding	13	<= 4 weeks
Haematochezia	13	<= 4 weeks
Haematoma muscle	13	<= 4 weeks
Haemorrhagic diathesis	13	<= 4 weeks
Localised oedema	13	<= 4 weeks
Myocardial necrosis marker increased	13	<= 4 weeks
Paresis	13	<= 4 weeks
Parosmia	13	<= 4 weeks
Productive cough	13	<= 4 weeks
Simple partial seizures	13	<= 4 weeks
Sleep talking	13	<= 4 weeks
Status epilepticus	13	<= 4 weeks
Tongue ulceration	13	<= 4 weeks
Urticaria	13	<= 4 weeks
Uterine haemorrhage	13	<= 4 weeks
Vasculitic rash	13	<= 4 weeks
Retrograde ejaculation	12.75	<= 4 weeks
Anal incontinence	12.5	<= 4 weeks
Arrhythmia	12.5	<= 4 weeks
Hepatic cytolysis	12.5	<= 4 weeks
Oedema	12.5	<= 4 weeks
Akinesia	12.25	<= 4 weeks
Angle closure glaucoma	12	<= 4 weeks
Cholestasis	12	<= 4 weeks
Cogwheel rigidity	12	<= 4 weeks
Coordination abnormal	12	<= 4 weeks
Extrapyramidal disorder	12	<= 4 weeks
Extrasystoles	12	<= 4 weeks
Eye movement disorder	12	<= 4 weeks
Glossodynia	12	<= 4 weeks
Pain of skin	12	<= 4 weeks
Reduced facial expression	12	<= 4 weeks
Respiratory tract infection	12	<= 4 weeks
Resting tremor	12	<= 4 weeks
Suicidal ideation	12	<= 4 weeks
Weight loss poor	12	<= 4 weeks
Tinnitus	11.75	<= 4 weeks
Cell death	11.5	<= 4 weeks
Cheilitis	11.5	<= 4 weeks
Gun shot wound	11.5	<= 4 weeks
Hypoglycaemia	11.5	<= 4 weeks
Mental status changes	11.5	<= 4 weeks
Mucosal inflammation	11.5	<= 4 weeks
Toxic epidermal necrolysis	11.5	<= 4 weeks
Disturbance in social behaviour	11.25	<= 4 weeks
Adjustment disorder with depressed mood	11	<= 4 weeks
Blood sodium decreased	11	<= 4 weeks
Cardio-respiratory arrest	11	<= 4 weeks
Cystitis	11	<= 4 weeks
Dissociative disorder	11	<= 4 weeks
Dyskinesia	11	<= 4 weeks
Dysphonia	11	<= 4 weeks
Epistaxis	11	<= 4 weeks
Exfoliative rash	11	<= 4 weeks
Gastritis erosive	11	<= 4 weeks
Herpes simplex	11	<= 4 weeks
Loss of personal independence in daily activities	11	<= 4 weeks
Male sexual dysfunction	11	<= 4 weeks
Nightmare	11	<= 4 weeks
Pain	11	<= 4 weeks
Prothrombin level decreased	11	<= 4 weeks
Restless legs syndrome	11	<= 4 weeks
Self-injurious ideation	11	<= 4 weeks
Thinking abnormal	11	<= 4 weeks
Toxic skin eruption	11	<= 4 weeks
Vaginal haemorrhage	11	<= 4 weeks
Erectile dysfunction	10.5	<= 4 weeks
Eye inflammation	10.5	<= 4 weeks
Gastrooesophageal reflux disease	10.5	<= 4 weeks
Neck pain	10.5	<= 4 weeks
Shock haemorrhagic	10.5	<= 4 weeks
Vulvovaginal discomfort	10.5	<= 4 weeks
Anosmia	10	<= 4 weeks
Apraxia	10	<= 4 weeks
Blood glucose decreased	10	<= 4 weeks
Body temperature fluctuation	10	<= 4 weeks
Derealisation	10	<= 4 weeks
Dermatitis	10	<= 4 weeks
Dermatitis exfoliative	10	<= 4 weeks
Disinhibition	10	<= 4 weeks
Disturbance in attention	10	<= 4 weeks
Gait disturbance	10	<= 4 weeks
Gastroenteritis	10	<= 4 weeks
General physical health deterioration	10	<= 4 weeks
Hypertensive crisis	10	<= 4 weeks
Hyponatraemia	10	<= 4 weeks
Inappropriate antidiuretic hormone secretion	10	<= 4 weeks
Muscle rigidity	10	<= 4 weeks
Muscle spasticity	10	<= 4 weeks
Neurological symptom	10	<= 4 weeks
Pain in extremity	10	<= 4 weeks
Prothrombin time prolonged	10	<= 4 weeks
Rhabdomyolysis	10	<= 4 weeks
Scar	10	<= 4 weeks
Stevens-Johnson syndrome	10	<= 4 weeks
Xerosis	10	<= 4 weeks
Behaviour disorder	9.5	<= 4 weeks
Formication	9.5	<= 4 weeks
Genital anaesthesia	9.5	<= 4 weeks
Hunger	9.5	<= 4 weeks
Musculoskeletal chest pain	9.5	<= 4 weeks
Myoclonus	9.5	<= 4 weeks
Electrocardiogram QT prolonged	9.104515	<= 4 weeks
Angioedema	9	<= 4 weeks
Bronchospasm	9	<= 4 weeks
Chorea	9	<= 4 weeks
Condition aggravated	9	<= 4 weeks
Ejaculation disorder	9	<= 4 weeks
Focal dyscognitive seizures	9	<= 4 weeks
Hypervolaemia	9	<= 4 weeks
Mobility decreased	9	<= 4 weeks
Mouth ulceration	9	<= 4 weeks
Muscle twitching	9	<= 4 weeks
Oliguria	9	<= 4 weeks
Peripheral swelling	9	<= 4 weeks
Respiratory arrest	9	<= 4 weeks
Therapeutic response increased	9	<= 4 weeks
Hangover	8.75	<= 4 weeks
Rhinorrhoea	8.75	<= 4 weeks
Abdominal pain lower	8.5	<= 4 weeks
Abnormal loss of weight	8.5	<= 4 weeks
Balance disorder	8.5	<= 4 weeks
Hypokalaemia	8.5	<= 4 weeks
Hyporeflexia	8.5	<= 4 weeks
Nocturia	8.5	<= 4 weeks
Urine odour abnormal	8.5	<= 4 weeks
Acute kidney injury	8	<= 4 weeks
Arrhythmia supraventricular	8	<= 4 weeks
Back pain	8	<= 4 weeks
Blister	8	<= 4 weeks
Decreased interest	8	<= 4 weeks
Dermatitis acneiform	8	<= 4 weeks
Dermatitis exfoliative generalised	8	<= 4 weeks
Drug eruption	8	<= 4 weeks
Emotional distress	8	<= 4 weeks
Exophthalmos	8	<= 4 weeks
Expired product administered	8	<= 4 weeks
Genital paraesthesia	8	<= 4 weeks
Gout	8	<= 4 weeks
Hypotonia	8	<= 4 weeks
Impaired work ability	8	<= 4 weeks
Laryngeal oedema	8	<= 4 weeks
Liver function test increased	8	<= 4 weeks
Mouth haemorrhage	8	<= 4 weeks
Myalgia	8	<= 4 weeks
Paraesthesia	8	<= 4 weeks
Psychomotor skills impaired	8	<= 4 weeks
Rash maculo-papular	8	<= 4 weeks
Rash papular	8	<= 4 weeks
Rash vesicular	8	<= 4 weeks
Swelling of eyelid	8	<= 4 weeks
Systemic lupus erythematosus rash	8	<= 4 weeks
Tonic clonic movements	8	<= 4 weeks
Chromaturia	7.5	<= 4 weeks
Palmar erythema	7.5	<= 4 weeks
Post procedural haemorrhage	7.5	<= 4 weeks
Rash morbilliform	7.5	<= 4 weeks
Tongue blistering	7.5	<= 4 weeks
Abnormal dreams	7	<= 4 weeks
Ageusia	7	<= 4 weeks
Anisocoria	7	<= 4 weeks
Apathy	7	<= 4 weeks
Ataxia	7	<= 4 weeks
Blepharitis	7	<= 4 weeks
Candida infection	7	<= 4 weeks
Drug ineffective	7	<= 4 weeks
Dysaesthesia	7	<= 4 weeks
Dystonia	7	<= 4 weeks
Ear pain	7	<= 4 weeks
Ejaculation failure	7	<= 4 weeks
Electrolyte imbalance	7	<= 4 weeks
Erection increased	7	<= 4 weeks
Glossitis	7	<= 4 weeks
Hallucination, auditory	7	<= 4 weeks
Hallucinations, mixed	7	<= 4 weeks
Head discomfort	7	<= 4 weeks
Heart rate decreased	7	<= 4 weeks
Hyperkinesia	7	<= 4 weeks
Hyperpyrexia	7	<= 4 weeks
Hyperreflexia	7	<= 4 weeks
Hypersomnia	7	<= 4 weeks
International normalised ratio increased	7	<= 4 weeks
Meniere's disease	7	<= 4 weeks
Micturition urgency	7	<= 4 weeks
Migraine	7	<= 4 weeks
Muscle contractions involuntary	7	<= 4 weeks
Muscle spasms	7	<= 4 weeks
Neuroleptic malignant syndrome	7	<= 4 weeks
Neurosis	7	<= 4 weeks
Opisthotonus	7	<= 4 weeks
Paranoia	7	<= 4 weeks
Pelvic pain	7	<= 4 weeks
Peripheral coldness	7	<= 4 weeks
Polyuria	7	<= 4 weeks
Pruritus	7	<= 4 weeks
Pruritus genital	7	<= 4 weeks
Rash	7	<= 4 weeks
Rash erythematous	7	<= 4 weeks
Sinus bradycardia	7	<= 4 weeks
Sleep disorder	7	<= 4 weeks
Speech disorder	7	<= 4 weeks
Stomatitis	7	<= 4 weeks
Syncope	7	<= 4 weeks
Therapeutic product effect decreased	7	<= 4 weeks
Tic	7	<= 4 weeks
Tongue spasm	7	<= 4 weeks
Toothache	7	<= 4 weeks
Vaginal discharge	7	<= 4 weeks
Vulvovaginitis	7	<= 4 weeks
Acute generalised exanthematous pustulosis	6.5	<= 4 weeks
Ear discomfort	6.5	<= 4 weeks
Lacrimation disorder	6.5	<= 4 weeks
Pleurothotonus	6.5	<= 4 weeks
Snoring	6.5	<= 4 weeks
Accommodation disorder	6	<= 4 weeks
Anorgasmia	6	<= 4 weeks
Antidiuretic hormone abnormality	6	<= 4 weeks
Burn oesophageal	6	<= 4 weeks
C-reactive protein increased	6	<= 4 weeks
Confusional state	6	<= 4 weeks
Disseminated intravascular coagulation	6	<= 4 weeks
Dissociation	6	<= 4 weeks
Dry eye	6	<= 4 weeks
Dysphemia	6	<= 4 weeks
Dyspnoea	6	<= 4 weeks
Ear disorder	6	<= 4 weeks
Eating disorder	6	<= 4 weeks
Eye pain	6	<= 4 weeks
Face oedema	6	<= 4 weeks
Facial paralysis	6	<= 4 weeks
Female orgasmic disorder	6	<= 4 weeks
Hallucination, visual	6	<= 4 weeks
Heart rate irregular	6	<= 4 weeks
Hiccups	6	<= 4 weeks
Hypochloraemia	6	<= 4 weeks
Illness	6	<= 4 weeks
Increased appetite	6	<= 4 weeks
Lethargy	6	<= 4 weeks
Libido increased	6	<= 4 weeks
Nasal congestion	6	<= 4 weeks
Ocular discomfort	6	<= 4 weeks
Orthostatic hypotension	6	<= 4 weeks
Panic attack	6	<= 4 weeks
Parkinsonian gait	6	<= 4 weeks
Photopsia	6	<= 4 weeks
Prostatic disorder	6	<= 4 weeks
Pyrexia	6	<= 4 weeks
Salivary hypersecretion	6	<= 4 weeks
Sedation complication	6	<= 4 weeks
Spinal fracture	6	<= 4 weeks
Suicidal behaviour	6	<= 4 weeks
Tongue disorder	6	<= 4 weeks
Urine flow decreased	6	<= 4 weeks
Vertigo	6	<= 4 weeks
Xerophthalmia	6	<= 4 weeks
Henoch-Schonlein purpura	5.5	<= 4 weeks
Ileus paralytic	5.5	<= 4 weeks
Pharyngitis	5.5	<= 4 weeks
Staring	5.25	<= 4 weeks
Abnormal faeces	5	<= 4 weeks
Acute psychosis	5	<= 4 weeks
Akathisia	5	<= 4 weeks
Asthenopia	5	<= 4 weeks
Atrial flutter	5	<= 4 weeks
Bruxism	5	<= 4 weeks
Cerebellar syndrome	5	<= 4 weeks
Constipation	5	<= 4 weeks
Delirium	5	<= 4 weeks
Diplopia	5	<= 4 weeks
Disorientation	5	<= 4 weeks
Dysuria	5	<= 4 weeks
Erythema	5	<= 4 weeks
Eye irritation	5	<= 4 weeks
Fear	5	<= 4 weeks
Female sexual dysfunction	5	<= 4 weeks
Gastritis	5	<= 4 weeks
Genital hypoaesthesia	5	<= 4 weeks
Hallucination	5	<= 4 weeks
Head titubation	5	<= 4 weeks
Hepatic pain	5	<= 4 weeks
Hypersensitivity	5	<= 4 weeks
Hyperthermia malignant	5	<= 4 weeks
Hypertonia	5	<= 4 weeks
Hypokinesia	5	<= 4 weeks
Immobile	5	<= 4 weeks
Laryngeal pain	5	<= 4 weeks
Logorrhoea	5	<= 4 weeks
Loss of consciousness	5	<= 4 weeks
Micturition disorder	5	<= 4 weeks
Musculoskeletal stiffness	5	<= 4 weeks
Mutism	5	<= 4 weeks
Oromandibular dystonia	5	<= 4 weeks
Oropharyngeal pain	5	<= 4 weeks
Penis disorder	5	<= 4 weeks
Periorbital oedema	5	<= 4 weeks
Presyncope	5	<= 4 weeks
Prothrombin time shortened	5	<= 4 weeks
Therapeutic response decreased	5	<= 4 weeks
Urinary retention	5	<= 4 weeks
Visual impairment	5	<= 4 weeks
Eye swelling	4.75	<= 4 weeks
Tension	4.75	<= 4 weeks
Blood pressure systolic increased	4.5	<= 4 weeks
Conversion disorder	4.5	<= 4 weeks
Dizziness postural	4.5	<= 4 weeks
Drug tolerance decreased	4.5	<= 4 weeks
Eyelid oedema	4.5	<= 4 weeks
Intrusive thoughts	4.5	<= 4 weeks
Laryngospasm	4.5	<= 4 weeks
Negative thoughts	4.5	<= 4 weeks
Rash pruritic	4.5	<= 4 weeks
Respiratory rate increased	4.5	<= 4 weeks
Swelling	4.5	<= 4 weeks
Breath odour	4.05	<= 4 weeks
Activation syndrome	4	<= 4 weeks
Anuria	4	<= 4 weeks
Anxiety disorder	4	<= 4 weeks
Blood pressure abnormal	4	<= 4 weeks
Body temperature increased	4	<= 4 weeks
Bone pain	4	<= 4 weeks
Bradycardia	4	<= 4 weeks
Chest pain	4	<= 4 weeks
Circulatory collapse	4	<= 4 weeks
Depressed mood	4	<= 4 weeks
Dermatitis allergic	4	<= 4 weeks
Diabetes mellitus inadequate control	4	<= 4 weeks
Drug ineffective for unapproved indication	4	<= 4 weeks
Dysarthria	4	<= 4 weeks
Dysphoria	4	<= 4 weeks
Fatigue	4	<= 4 weeks
Gait inability	4	<= 4 weeks
Gastrointestinal disorder	4	<= 4 weeks
Hyperhidrosis	4	<= 4 weeks
Hypertension	4	<= 4 weeks
Hyperthermia	4	<= 4 weeks
Hypoaesthesia	4	<= 4 weeks
Irritable bowel syndrome	4	<= 4 weeks
Lip swelling	4	<= 4 weeks
Movement disorder	4	<= 4 weeks
Muscular weakness	4	<= 4 weeks
Panic disorder	4	<= 4 weeks
Panic reaction	4	<= 4 weeks
Papule	4	<= 4 weeks
Paralysis	4	<= 4 weeks
Photophobia	4	<= 4 weeks
Pollakiuria	4	<= 4 weeks
Psychomotor hyperactivity	4	<= 4 weeks
Rash macular	4	<= 4 weeks
Sciatica	4	<= 4 weeks
Sensation of foreign body	4	<= 4 weeks
Skin reaction	4	<= 4 weeks
Skin warm	4	<= 4 weeks
Taste disorder	4	<= 4 weeks
Testicular pain	4	<= 4 weeks
Vision blurred	4	<= 4 weeks
Dysstasia	3.75069	<= 4 weeks
Epigastric discomfort	3.75	<= 4 weeks
Thirst	3.55	<= 4 weeks
Bedridden	3.5	<= 4 weeks
Daydreaming	3.5	<= 4 weeks
Hyperphagia	3.5	<= 4 weeks
Inhibitory drug interaction	3.5	<= 4 weeks
Neuralgia	3.5	<= 4 weeks
Peripheral ischaemia	3.5	<= 4 weeks
Tongue coated	3.5	<= 4 weeks
Tourette's disorder	3.5	<= 4 weeks
Urinary hesitation	3.5	<= 4 weeks
Therapeutic response unexpected	3.05	<= 4 weeks
Abdominal distension	3	<= 4 weeks
Acidosis	3	<= 4 weeks
Adverse reaction	3	<= 4 weeks
Agitation	3	<= 4 weeks
Anxiety	3	<= 4 weeks
Asthenia	3	<= 4 weeks
Autonomic nervous system imbalance	3	<= 4 weeks
Blood pressure increased	3	<= 4 weeks
Convulsions local	3	<= 4 weeks
Decreased appetite	3	<= 4 weeks
Drug hypersensitivity	3	<= 4 weeks
Drug interaction	3	<= 4 weeks
Dysgeusia	3	<= 4 weeks
Dysphagia	3	<= 4 weeks
Euphoric mood	3	<= 4 weeks
Eye pruritus	3	<= 4 weeks
Faeces soft	3	<= 4 weeks
Feeling jittery	3	<= 4 weeks
Foaming at mouth	3	<= 4 weeks
Frequent bowel movements	3	<= 4 weeks
Hyperventilation	3	<= 4 weeks
Hypoaesthesia oral	3	<= 4 weeks
Illusion	3	<= 4 weeks
Incoherent	3	<= 4 weeks
Joint stiffness	3	<= 4 weeks
Limb discomfort	3	<= 4 weeks
Lip dry	3	<= 4 weeks
Lip oedema	3	<= 4 weeks
Mouth swelling	3	<= 4 weeks
Muscle tightness	3	<= 4 weeks
Musculoskeletal pain	3	<= 4 weeks
Nystagmus	3	<= 4 weeks
Ocular hyperaemia	3	<= 4 weeks
Oedema mouth	3	<= 4 weeks
Pain in jaw	3	<= 4 weeks
Peripheral vascular disorder	3	<= 4 weeks
Phobia	3	<= 4 weeks
Polydipsia	3	<= 4 weeks
Psychomotor retardation	3	<= 4 weeks
Pustule	3	<= 4 weeks
Rectal tenesmus	3	<= 4 weeks
Respiration abnormal	3	<= 4 weeks
Sensory loss	3	<= 4 weeks
Serotonin syndrome	3	<= 4 weeks
Skin burning sensation	3	<= 4 weeks
Sputum increased	3	<= 4 weeks
Tetany	3	<= 4 weeks
Therapeutic response changed	3	<= 4 weeks
Tonic convulsion	3	<= 4 weeks
Tremor	3	<= 4 weeks
Trismus	3	<= 4 weeks
Tunnel vision	3	<= 4 weeks
Type IV hypersensitivity reaction	3	<= 4 weeks
Urine output decreased	3	<= 4 weeks
Wheezing	3	<= 4 weeks
Feeling abnormal	2.75	<= 4 weeks
Cardiac discomfort	2.5	<= 4 weeks
Lacrimation increased	2.5	<= 4 weeks
Metabolic acidosis	2.5	<= 4 weeks
Oculogyric crisis	2.5	<= 4 weeks
Unresponsive to stimuli	2.5	<= 4 weeks
Abdominal discomfort	2	<= 4 weeks
Abdominal pain	2	<= 4 weeks
Adverse drug reaction	2	<= 4 weeks
Amblyopia	2	<= 4 weeks
Anaphylactic shock	2	<= 4 weeks
Anaphylactoid reaction	2	<= 4 weeks
Anorectal disorder	2	<= 4 weeks
Antisocial behaviour	2	<= 4 weeks
Appetite disorder	2	<= 4 weeks
Attention deficit hyperactivity disorder	2	<= 4 weeks
Binge eating	2	<= 4 weeks
Blood pressure decreased	2	<= 4 weeks
Blood pressure fluctuation	2	<= 4 weeks
Burning sensation	2	<= 4 weeks
Chest discomfort	2	<= 4 weeks
Clonus	2	<= 4 weeks
Cold sweat	2	<= 4 weeks
Communication disorder	2	<= 4 weeks
Depersonalisation/derealisation disorder	2	<= 4 weeks
Diarrhoea	2	<= 4 weeks
Discomfort	2	<= 4 weeks
Dizziness	2	<= 4 weeks
Dry mouth	2	<= 4 weeks
Dyspepsia	2	<= 4 weeks
Facial pain	2	<= 4 weeks
Feeding disorder	2	<= 4 weeks
Feeling cold	2	<= 4 weeks
Feeling hot	2	<= 4 weeks
Feeling of body temperature change	2	<= 4 weeks
Flatulence	2	<= 4 weeks
Flight of ideas	2	<= 4 weeks
Flushing	2	<= 4 weeks
Gastrointestinal motility disorder	2	<= 4 weeks
Gastrointestinal pain	2	<= 4 weeks
Headache	2	<= 4 weeks
Hot flush	2	<= 4 weeks
Hyperchlorhydria	2	<= 4 weeks
Hypopnoea	2	<= 4 weeks
Hypoventilation	2	<= 4 weeks
Initial insomnia	2	<= 4 weeks
Insomnia	2	<= 4 weeks
Malaise	2	<= 4 weeks
Merycism	2	<= 4 weeks
Narcolepsy	2	<= 4 weeks
Nervousness	2	<= 4 weeks
Oral pain	2	<= 4 weeks
Pallor	2	<= 4 weeks
Palpitations	2	<= 4 weeks
Paraesthesia oral	2	<= 4 weeks
Pharyngeal oedema	2	<= 4 weeks
Pneumonia aspiration	2	<= 4 weeks
Poor quality sleep	2	<= 4 weeks
Restlessness	2	<= 4 weeks
Shock	2	<= 4 weeks
Sinus tachycardia	2	<= 4 weeks
Stupor	2	<= 4 weeks
Suffocation feeling	2	<= 4 weeks
Swelling face	2	<= 4 weeks
Swollen tongue	2	<= 4 weeks
Terminal insomnia	2	<= 4 weeks
Therapy change	2	<= 4 weeks
Throat tightness	2	<= 4 weeks
Tongue discomfort	2	<= 4 weeks
Tongue oedema	2	<= 4 weeks
Tongue paralysis	2	<= 4 weeks
Yawning	2	<= 4 weeks
Body temperature decreased	1.5	<= 4 weeks
Electrocardiogram QT interval abnormal	1.5	<= 4 weeks
Feeling drunk	1.5	<= 4 weeks
Maternal exposure during breast feeding	1.5	<= 4 weeks
Pupillary reflex impaired	1.5	<= 4 weeks
Tachyphrenia	1.5	<= 4 weeks
Urinary tract disorder	1.5	<= 4 weeks
Abdominal pain upper	1.25	<= 4 weeks
Accidental overdose	1	<= 4 weeks
Altered state of consciousness	1	<= 4 weeks
Anaphylactic reaction	1	<= 4 weeks
Anticholinergic syndrome	1	<= 4 weeks
Bladder pain	1	<= 4 weeks
Bradykinesia	1	<= 4 weeks
Brain fog	1	<= 4 weeks
Cardiac flutter	1	<= 4 weeks
Central nervous system stimulation	1	<= 4 weeks
Change of bowel habit	1	<= 4 weeks
Chills	1	<= 4 weeks
Choking sensation	1	<= 4 weeks
Depressed level of consciousness	1	<= 4 weeks
Drug intolerance	1	<= 4 weeks
Electrocardiogram QRS complex prolonged	1	<= 4 weeks
Eructation	1	<= 4 weeks
Facial spasm	1	<= 4 weeks
Gastric dilatation	1	<= 4 weeks
Heart rate increased	1	<= 4 weeks
Hypervigilance	1	<= 4 weeks
Hypotension	1	<= 4 weeks
Hypothermia	1	<= 4 weeks
Injection site inflammation	1	<= 4 weeks
Jaw disorder	1	<= 4 weeks
Labelled drug-drug interaction medication error	1	<= 4 weeks
Lactation insufficiency	1	<= 4 weeks
Lactic acidosis	1	<= 4 weeks
Listless	1	<= 4 weeks
Middle insomnia	1	<= 4 weeks
Morbid thoughts	1	<= 4 weeks
Myasthenic syndrome	1	<= 4 weeks
Mydriasis	1	<= 4 weeks
Nausea	1	<= 4 weeks
Oesophageal pain	1	<= 4 weeks
Oral discomfort	1	<= 4 weeks
Periorbital swelling	1	<= 4 weeks
Persistent genital arousal disorder	1	<= 4 weeks
Pharyngeal swelling	1	<= 4 weeks
Product substitution issue	1	<= 4 weeks
Prothrombin time ratio decreased	1	<= 4 weeks
Retching	1	<= 4 weeks
Sedation	1	<= 4 weeks
Somnolence	1	<= 4 weeks
Tachycardia	1	<= 4 weeks
Throat irritation	1	<= 4 weeks
Toxic encephalopathy	1	<= 4 weeks
Vaginal infection	1	<= 4 weeks
Vasodilatation	1	<= 4 weeks
Vomiting	1	<= 4 weeks
Acute respiratory failure	0.75	<= 4 weeks
Potentiating drug interaction	0.625	<= 4 weeks
Enteritis	0.5	<= 4 weeks
Feeling of relaxation	0.5	<= 4 weeks
Paradoxical drug reaction	0.5	<= 4 weeks
Pupil fixed	0.5	<= 4 weeks
Self-destructive behaviour	0.5	<= 4 weeks
Cardiogenic shock	0.125	<= 4 weeks
Areflexia	0.05243	<= 4 weeks
Accidental exposure to product	0	<= 4 weeks
Accidental exposure to product by child	0	<= 4 weeks
Alcohol abuse	0	<= 4 weeks
Alcohol poisoning	0	<= 4 weeks
Anaesthesia oral	0	<= 4 weeks
Blood pressure systolic decreased	0	<= 4 weeks
Bradyphrenia	0	<= 4 weeks
Bradypnoea	0	<= 4 weeks
Clumsiness	0	<= 4 weeks
Coma	0	<= 4 weeks
Coma scale abnormal	0	<= 4 weeks
Contraindicated product administered	0	<= 4 weeks
Drug abuse	0	<= 4 weeks
Extra dose administered	0	<= 4 weeks
Fear of death	0	<= 4 weeks
Functional gastrointestinal disorder	0	<= 4 weeks
Gastrointestinal sounds abnormal	0	<= 4 weeks
Inappropriate schedule of product administration	0	<= 4 weeks
Incorrect dosage administered	0	<= 4 weeks
Incorrect dose administered	0	<= 4 weeks
Incorrect drug administration rate	0	<= 4 weeks
Incorrect route of product administration	0	<= 4 weeks
Injection site pain	0	<= 4 weeks
Injection site swelling	0	<= 4 weeks
Intentional overdose	0	<= 4 weeks
Intentional product misuse	0	<= 4 weeks
Intentional product use issue	0	<= 4 weeks
Intentional self-injury	0	<= 4 weeks
Medication error	0	<= 4 weeks
Miosis	0	<= 4 weeks
No adverse event	0	<= 4 weeks
Oesophageal disorder	0	<= 4 weeks
Off label use	0	<= 4 weeks
Oropharyngeal discomfort	0	<= 4 weeks
Overdose	0	<= 4 weeks
Poisoning	0	<= 4 weeks
Poisoning deliberate	0	<= 4 weeks
Prescribed overdose	0	<= 4 weeks
Product administration error	0	<= 4 weeks
Product dispensing error	0	<= 4 weeks
Product prescribing error	0	<= 4 weeks
Product quality issue	0	<= 4 weeks
Product taste abnormal	0	<= 4 weeks
Product use in unapproved indication	0	<= 4 weeks
Product use issue	0	<= 4 weeks
Respiratory acidosis	0	<= 4 weeks
Respiratory depression	0	<= 4 weeks
Respiratory rate decreased	0	<= 4 weeks
Slow response to stimuli	0	<= 4 weeks
Slow speech	0	<= 4 weeks
Sluggishness	0	<= 4 weeks
Sopor	0	<= 4 weeks
Substance abuse	0	<= 4 weeks
Suicide attempt	0	<= 4 weeks
Systolic hypertension	0	<= 4 weeks
Tobacco user	0	<= 4 weeks
Toxicity to various agents	0	<= 4 weeks
Underdose	0	<= 4 weeks
Wrong patient	0	<= 4 weeks
Wrong patient received product	0	<= 4 weeks
Wrong product administered	0	<= 4 weeks
Wrong technique in product usage process	0	<= 4 weeks

## References

[1] Chu A, Wadhwa R (2025). Selective serotonin reuptake inhibitors. StatPearls [Internet].

[2] Strawn JR, Mills JA, Poweleit EA, Ramsey LB, Croarkin PE (2023). Adverse effects of antidepressant medications and their management in children and adolescents. Pharmacotherapy.

[3] Hu XH, Bull SA, Hunkeler EM (2004). Incidence and duration of side effects and those rated as bothersome with selective serotonin reuptake inhibitor treatment for depression: patient report versus physician estimate. J Clin Psychiatry.

[4] Bull SA, Hunkeler EM, Lee JY (2002). Discontinuing or switching selective serotonin-reuptake inhibitors. Ann Pharmacother.

[5] Wakao R, Taavola H, Sandberg L (2019). Data-driven identification of adverse event reporting patterns for Japan in VigiBase, the WHO global database of individual case safety reports. Drug Saf.

[6] Cho H, Lee K, Wang SM (2025). Global burden of antidepressant-associated seizures from 1967 to 2023: a comparative analysis of the international pharmacovigilance database. J Affect Disord.

[7] Zhao Y, Zhang Y, Yang L, Zhang K, Li S (2024). Safety profile of selective serotonin reuptake inhibitors in real-world settings: a pharmacovigilance study based on the FDA Adverse Event Reporting System. Ann Pharmacother.

[8] Yu Q, Yao J, Li E (2025). Neurological adverse events associated with antidepressants: a comprehensive 22-year analysis of the FDA adverse event reporting system. Front Pharmacol.

[9] Shostak ES, Lang JM, Quinn WK (2025). Effects of selective serotonin reuptake inhibitor (SSRI) use on cardiometabolic health and risk in young healthy individuals: a preliminary matched pairs study. Physiol Rep.

[10] Valeiro C, Matos C, Scholl J, van Hunsel F (2022). Drug-induced sexual dysfunction: an analysis of reports to a national pharmacovigilance database. Drug Saf.

[11] Cao J, Chen Z, Wang Y (2024). Overweight and glucose/lipid metabolism abnormality associated with SSRIs: a pharmacovigilance study based on the FDA adverse event reporting system. Front Pharmacol.

[12] Bala A, Nguyen HM, Hellstrom WJ (2018). Post-SSRI sexual dysfunction: a literature review. Sex Med Rev.

[13] Studt A, Gannon M, Orzel J, Vaughan A, Pearlman AM (2021). Characterizing post-SSRI sexual dysfunction and its impact on quality of life through an international online survey. Int J Risk Saf Med.

[14] Ben-Sheetrit J, Hermon Y, Birkenfeld S, Gutman Y, Csoka AB, Toren P (2023). Estimating the risk of irreversible post-SSRI sexual dysfunction (PSSD) due to serotonergic antidepressants. Ann Gen Psychiatry.

[15] Wadhwa R, Kumar M, Talegaonkar S, Vohora D (2017). Serotonin reuptake inhibitors and bone health: a review of clinical studies and plausible mechanisms. Osteoporos Sarcopenia.

[16] Edinoff AN, Akuly HA, Hanna TA (2021). Selective serotonin reuptake inhibitors and adverse effects: a narrative review. Neurol Int.

[17] O'Connell NS, Zhao F, Lee JW (2024). Importance of low- and moderate-grade adverse events in patients' treatment experience and treatment discontinuation: an analysis of the E1912 trial. J Clin Oncol.

[18] Panteli D, Legido-Quigley H, Reichebner C, Ollenschläger G, Schäfer C, Busse R (2019). Improving Healthcare Quality in Europe: Characteristics, Effectiveness and Implementation of Different Strategies. Improving healthcare quality in Europe: Characteristics, effectiveness and implementation of different strategies [Internet].

[19] Olguner Eker Ö, Özsoy S, Eker B, Doğan H (2017). Metabolic effects of antidepressant treatment. Noro Psikiyatr Ars.

[20] Pilipiec P, Liwicki M, Bota A (2022). Using machine learning for pharmacovigilance: a systematic review. Pharmaceutics.

[21] Sharew NT, Clark SR, Schubert KO, Amare AT (2024). Pharmacogenomic scores in psychiatry: systematic review of current evidence. Transl Psychiatry.

[22] Lee J, Chang SM (2022). Confounding by indication in studies of selective serotonin reuptake inhibitors. Psychiatry Investig.

[23] Jafari E, Blackman MH, Karnes JH (2024). Using electronic health records for clinical pharmacology research: challenges and considerations. Clin Transl Sci.

[24] Kim JV, Davis SE, Matheny ME, Smith JC (2024). Integrating electronic health records with other data sources for postmarket drug safety signal identification: a review. Front Drug Saf Regul.

